# Relato seletivo de reações adversas renais relacionadas aos inibidores seletivos da ciclo-oxigenase-2 em ensaios clínicos randomizados: estudo transversal, 2023

**DOI:** 10.1590/S2237-96222026v35e20260191.pt

**Published:** 2026-11-09

**Authors:** Tayanny Margarida Menezes Almeida Biase, Marcus Tolentino Silva, Tais Freire Galvão

**Affiliations:** 1 Universidade Estadual de Campinas, Faculdade de Ciências Farmacêuticas, Campinas, SP, Brasil; 2 Universidade de Brasília, Departamento de Saúde Coletiva, Brasília, DF, Brasil

**Keywords:** Viés de Publicação, Revisão por Pares, Evento Adverso, Inibidores de Ciclo-Oxigenase 2, Estudos Transversais, Sesgo de Publicación, Revisión por Pares, Evento Adverso, Inhibidores de la Ciclooxigenasa 2, Estudios Transversales

## Abstract

**Objetivo:**

Identificar frequência de relato seletivo de desfechos em ensaios clínicos randomizados que avaliaram a segurança renal de inibidores seletivos da ciclo-oxigenase-2 (coxibs) e explorar fatores associados à sua ocorrência.

**Métodos:**

Trata-se de análise transversal baseada em revisão sistemática prévia sobre efeitos renais dos coxibs. Considerou-se como relato seletivo de desfecho se os estudos apresentassem resultados somente de eventos adversos pré-definidos ou se adotassem limiar de incidência para relatar reação renal. Coletaram-se fator de impacto, duração da revisão por pares, local do estudo, tipo de coxib e fontes de financiamento. Variáveis contínuas foram descritas por média e por desvio padrão e categóricas por frequências. Fatores associados ao relato seletivo foram testados por teste qui-quadrado ou teste t (p-valor<0,05).

**Resultados:**

Dos 49 estudos incluídos, 30 apresentaram relato seletivo; 19 estabeleceram limiares para relatar reações e 11 relataram apenas eventos pré-especificados. A omissão de reações adversas foi mais frequente em periódicos com menor fator de impacto (n=29; 6,1**±**6,5). A revisão por pares foi maior em ensaios sem o viés de relato (n=11) do que naqueles com viés (n=15), porém sem significância (p-valor 0,470). Estudos sobre osteoartrite mostraram maior frequência de relato seletivo (20/26 versus 10/23; p-valor 0,016) e ensaios com duração ≤6 semanas apresentaram maior proporção desse viés (17/22) em comparação aos de maior duração (13/27; p-valor 0,037). A maioria dos estudos (41/49) foi financiada pela indústria farmacêutica.

**Conclusões:**

O relato seletivo de reações adversas renais foi comum em ensaios clínicos sobre coxibs, potencialmente influenciado por políticas editoriais e patrocínio da pesquisa.

## RESUMEN

**Objetivo:**

Identificar la frecuencia del registro selectivo de resultados en ensayos clínicos aleatorizados que evalúan la seguridad renal de los inhibidores selectivos de la ciclooxigenasa-2 (coxibs) y explorar los factores asociados a su aparición.

**Métodos:**

Análisis transversal basado en una revisión sistemática previa sobre los efectos renales de los coxibs. Se definió el registro selectivo de resultados como aquellos casos en los que los estudios presentaban resultados únicamente para eventos adversos predefinidos o aplicaban un umbral de incidencia para notificar reacciones renales. Se recopilaron datos sobre el factor de impacto, la duración de la revisión por pares, la ubicación del estudio, el tipo de coxib y las fuentes de financiación. Las variables continuas se describieron mediante la media y la desviación estándar, mientras que las variables categóricas se describieron mediante frecuencias. Los factores asociados al registro selectivo se evaluaron utilizando la prueba de chi-cuadrado o la prueba t (valor-p<0,05).

**Resultados:**

De los 49 estudios incluidos, 30 presentaron registro selectivo; 19 establecieron umbrales para notificar reacciones y 11 notificaron únicamente eventos preespecificados. La omisión de reacciones adversas fue más frecuente en revistas con factores de impacto más bajos (n=29; 6,1±6,5). La duración de la revisión por pares fue mayor en los ensayos sin sesgo (n=11) que en aquellos con sesgo (n=15), aunque no de forma significativa (valor-p 0,470). Los estudios sobre osteoartritis mostraron una mayor frecuencia de registro selectivo (20/26 frente a 10/23; valor-p 0,016), y los ensayos con una duración ≤6 semanas presentaron una mayor proporción de sesgo (17/22) en comparación con aquellos de mayor duración (13/27; valor-p 0,037). La mayoría de los estudios (41/49) fueron financiados por la industria.

**Conclusiones:**

El registro selectivo de reacciones adversas renales fue frecuente en los ensayos clínicos de coxibs, posiblemente influida por las políticas editoriales y el patrocinio de la investigación.

## Introdução

Os anti-inflamatórios inibidores seletivos da ciclo-oxigenase-2 (coxibs) foram introduzidos no mercado na década de 1990 com a promessa de reduzir a toxicidade gastrointestinal em comparação aos anti-inflamatórios não esteroides tradicionais [1]. Logo após seu uso generalizado, surgiram preocupações quanto à segurança cardiovascular [2], o que levou, em 2004, à retirada do mercado do rofecoxibe, o primeiro coxib a ser descontinuado por esse motivo [3, 4]. Esse processo tornou-se emblemático por questões relacionadas à transparência no relato de eventos adversos. Em um estudo randomizado que comparou rofecoxibe e naproxeno em pacientes com artrite reumatoide [5], a publicação original não apresentou integralmente dados cardiovasculares relevantes [6]. Posteriormente, uma nota de preocupação foi publicada e apontou que três casos adicionais de infarto do miocárdio, todos no grupo rofecoxibe, não haviam sido incluídos nos dados submetidos ao periódico, o que reforçou a importância do relato completo de eventos adversos em ensaios clínicos [6].

Essas preocupações com a segurança resultaram em restrições regulatórias, estratégias de comunicação de riscos e diretrizes de prescrição com o objetivo de limitar o uso inadequado [7]. Apesar dessas medidas com vistas ao uso racional, o uso de coxibs permaneceu crescente no Brasil [8] e em diferentes contextos, particularmente em populações com multimorbidade e com dor crônica [9].

Revisão sistemática de ensaios clínicos sobre coxibs observou que esses medicamentos aumentam o risco de efeitos adversos renais, inclusive hipertensão e edema, com certeza de evidência moderada a baixa [10]. O relato seletivo desses eventos adversos foi frequente, o que contribuiu para o risco de viés e de imprecisão dos resultados [10]. A omissão de reações adversas a medicamentos nas publicações pode induzir médicos e formuladores de políticas ao erro, com subestimação dos possíveis danos e criação de falsa sensação de segurança, o que pode resultar em maior exposição dos pacientes a danos graves [11]. O relato seletivo de desfechos também pode afetar diretamente a tomada de decisões clínicas e as políticas de saúde pública, especialmente em avaliações de segurança de medicamentos [12, 13]. Essa prática tem implicações diretas para sistemas de saúde universais, como o Sistema Único de Saúde (SUS), que depende de evidências robustas e completas para decisões de incorporação tecnológica, atualização de protocolos clínicos e monitoramento de segurança pós-comercialização [14].

Identificar os fatores associados ao viés de relato pode contribuir para o debate sobre a transparência e a confiabilidade das evidências clínicas para tomada de decisão em saúde. Nesse contexto, este estudo teve como objetivo identificar a frequência de relato seletivo de reações adversas renais e explorar características dos estudos associadas a ocorrência desse desfecho em ensaios clínicos incluídos em revisão sistemática que avaliou os efeitos renais dos coxibs.

## Métodos

### Desenho do estudo e participantes

Trata-se de análise transversal baseada em revisão sistemática prévia que avaliou os efeitos adversos renais dos coxibs [10]. Os ensaios clínicos randomizados incluídos na revisão sistemática foram elegíveis para esta análise [10].

### Contexto

A revisão sistemática seguiu protocolo registrado no International Prospective Register of Systematic Reviews (CRD42022380227). A revisão baseou-se em ensaios clínicos randomizados que avaliaram os efeitos renais dos coxibs (celecoxibe, etoricoxibe, lumiracoxibe, parecoxibe e valdecoxibe) em comparação com placebos. As buscas nas bases de dados foram realizadas até março de 2024, com estratégia de busca revisada por pares. Dois revisores independentes realizaram a seleção dos estudos, a extração de dados e a avaliação do risco de viés. Meta-análise, metarregressão e avaliação da certeza da evidência também foram conduzidas [10].

### Variáveis, fontes de dados e mensuração

A variável dependente deste estudo foi o relato seletivo de desfechos, dicotomizado como presente ou ausente, definido como a omissão, a apresentação incompleta ou a descrição insuficiente de reações adversas renais previamente avaliadas ou esperadas no contexto dos ensaios clínicos, o que dificulta a interpretação do perfil de segurança dos medicamentos. O relato seletivo de desfecho foi classificado como presente nos estudos com alto risco de viés no item nº 6 da ferramenta Cochrane Risk of Bias [15]. Os estudos foram classificados como alto risco de viés nesse domínio se: (i) adotaram um limiar de incidência para relatar eventos adversos (definiram frequência mínima do evento para relatar as reações adversas na publicação final) ou (ii) relataram apenas eventos adversos pré-especificados, com omissão de outros eventos adversos ocorridos durante o ensaio, caso esses não estivessem na lista inicial. Os estudos que não atenderam a esses critérios foram classificados como sem relato seletivo.

Foram coletadas as seguintes informações: nome do periódico, data de publicação, local do estudo, duração (em semanas), tipo de coxib, condição clínica dos participantes, número de participantes e indústria farmacêutica que fabricava os coxibs no momento da publicação dos estudos. As datas de submissão e de aceite foram obtidas no texto completo dos artigos ou nos websites dos periódicos para calcular o tempo de revisão por pares (em dias). Para maior clareza na comunicação, consideramos o tempo desde a submissão até o aceite como duração da revisão por pares. O fator de impacto dos periódicos (Journal Impact Factor) de 2022 e a frequência de acesso aberto ouro (*gold open access*) dos periódicos onde os estudos foram publicados foram obtidos do Journal Citation Report (Clarivate) em setembro de 2023.

Os dados da investigação estão disponíveis integralmente no Repositório de Dados de Pesquisa da Unicamp [16]. 

### Métodos estatísticos

Foram calculadas estatísticas descritivas, com inclusão de frequências, médias e desvio padrão (DP). Os fatores associados ao relato seletivo foram avaliados por meio do teste do qui-quadrado de Pearson para variáveis categóricas e o teste t de Student para variáveis contínuas, com a consideração de p-valor<0,05 como nível de significância. Todas as análises foram realizadas no software Stata, versão 14.2 (College Station, TX).

## Resultados

O relato seletivo foi observado em 30 dos 49 estudos incluídos na revisão sistemática. Os estudos foram conduzidos na América do Norte (n=35) e na Europa (n=14). A maioria foi publicada em revistas com fator de impacto (n=47); 31 estudos entre 1999-2006; 14 em periódicos de reumatologia e 10 em periódicos de medicina geral (Tabela 1 e Tabela suplementar 1).

O principal motivo de relato seletivo foi o uso de limiares de incidência para relatar reações adversas (n=19), com estabelecimento de frequência mínima de 1-2% (n=4), 3% (n=8) ou 4-5% (n=7) para relatar a ocorrência de reações adversas. 11 estudos relataram apenas eventos adversos pré-especificados (Tabela suplementar 2).

Foi possível calcular o tempo de processamento editorial em 25 estudos. Estudos sem viés de relato (n=11) tiveram maior duração da revisão por pares, embora não significativa (190,7±155, 3 dias), do que aqueles com esse viés (n=15; 155,1±91, 4 dias; p-valor 0,470). O número de participantes também foi maior em estudos sem viés de relato (n=19; 671,7±540,7) em comparação com ensaios com viés (n=30; 527,2±452,6; p-valor 0,317). O relato seletivo foi significativamente mais frequente em periódicos com menor fator de impacto (n=29; 6,1±6,5) em comparação com aqueles de maior prestígio (n=18; 29,0±54,5; p-valor 0,029). Não foram observadas diferenças significativas quanto ao acesso aberto, à data de publicação ou ao tipo de coxib (p-valor >0,05) (Tabela 2).

**Tabela 1:** Frequência das características dos ensaios clínicos com inibidores seletivos da ciclo-oxigenase-2 (coxibs) de acordo com a ocorrência de relato seletivo de reações adversas renais (n=49)

Variável	Com relato seletivo (n=30)	Sem relato seletivo (n=19)	p-valor^a^
Data de publicação			0,551
1999-2006	18	13	
2007-2018	12	6	
Local do estudo			0,354
América do Norte	20	15	
Europa	10	4	
Tipo de coxib			
Celecoxibe	16	9	0,684
Etoricoxibe	8	9	0,138
Lumiracoxibe	7	1	0,095
Parecoxibe	3	1	0,555
Valdecoxibe	2	0	0,250
Condição clínica			0,016
Osteoartrite	20	6	
Outros	10	13	
Duração do estudo (semanas)			0,037
<6	17	5	
>6	13	14	
Indústria farmacêutica			
Merck	22	8	0,029
Pfizer	23	14	0,813
Novartis	24	17	0,382

a Teste do qui-quadrado de Pearson.

**Tabela 2:** Média e desvio padrão (DP) das características dos ensaios clínicos de acordo com a ocorrência de relato seletivo de reações adversas renais (n=49)

Variável	Com relato seletivo		Sem relato seletivo		p-valor^a^
	n	Média±DP	n	Média±DP	
Fator de impacto	29	6,1±6,5	18	29,0±54,5	0,029
Porcentagem de acesso aberto	27	33,1±28,9	18	36,1±34,5	0,773
Número de participantes	30	527,2±452,6	19	671,7±540,7	0,317
Duração da revisão por pares (dias)	15	155,1±91,4	11	190,7±155,3	0,470

a Teste t de Student

Os estudos relacionados à osteoartrite mostraram maior frequência de relato seletivo (20/26 versus 10/23; p-valor 0,016). De modo semelhante, os ensaios com duração de até seis semanas apresentaram maior proporção de relato seletivo (17/22) em comparação com os de maior duração (13/27; p-valor 0,037).

A maioria dos estudos (41/49) recebeu financiamento da indústria farmacêutica que fabricava os coxibs no momento da publicação. As principais fabricantes que financiaram os ensaios foram Merck (n=19), Pfizer (n=12) e Novartis (n=8). Os estudos financiados pela Merck apresentaram menor frequência de relato seletivo (8/19 vs. 22/30; p-valor 0,029) (Tabela 1).

## Discussão

O relato seletivo de reações renais foi elevado em ensaios clínicos randomizados que avaliaram coxibs. O viés de relato foi mais comum em estudos publicados em periódicos com menor fator de impacto, em ensaios de curta duração e em pesquisas com envolvimento de pacientes com osteoartrite. O financiamento da indústria farmacêutica foi predominante entre os estudos incluídos, com variações entre os patrocinadores quanto à ocorrência de relato seletivo. 

Esta análise baseou-se em revisão sistemática previamente publicada [10], cujos critérios de elegibilidade, processo de seleção e de extração de dados por revisores independentes e avaliação do risco de viés influenciam diretamente os resultados obtidos. Possíveis limitações metodológicas da revisão original podem impactar a presente análise. O tempo entre a submissão e o aceite dos artigos foi considerado como tempo de revisão por pares, e não como uma medida da qualidade da revisão por pares. Essa variável pode ser influenciada por fatores não acessíveis aos autores, como etapas administrativas internas, tempo de resposta dos autores às solicitações de revisão, número de rodadas de revisão, disponibilidade de revisores e práticas editoriais específicas de cada periódico. Fatores como o contexto regulatório à época da publicação, a possível influência de revisores por pares e editores e as práticas editoriais específicas de cada periódico não foram avaliados. Esses aspectos podem desempenhar papel importante na ocorrência de relato seletivo e sua ausência nesta análise pode ter limitado a interpretação dos achados [17,18].

A alta frequência de relato seletivo de desfechos observada neste estudo é consistente com achados de outras áreas da pesquisa em saúde. Estudo de 2024 que analisou ensaios clínicos sobre o uso de antibióticos em cirurgias de terceiros molares, ao comparar os protocolos registrados com os artigos publicados, identificou relato seletivo de desfechos em 88% dos estudos [19]. De forma semelhante, revisão sistemática de ensaios clínicos publicados em periódicos de hematologia identificou relato seletivo de desfechos em grande proporção dos ensaios, com discrepâncias principalmente relacionadas à omissão, à adição ou à alteração dos desfechos primários [20]. 

Os resultados deste estudo destacaram a necessidade de melhorias estruturais para promover maior transparência e confiabilidade na comunicação científica. Para enfrentar esses desafios e aprimorar a transparência na ciência, diversas medidas são recomendadas, com inclusão do fortalecimento dos requisitos para o registro prospectivo de ensaios clínicos, da publicação completa dos dados de eventos adversos, independentemente da frequência observada, e da adesão a guias de redação científica, como o Consolidated Standards of Reporting Trials (CONSORT) [21]. O aprimoramento do processo de revisão por pares também é relevante, com maior capacitação de revisores e de editores para detectar práticas de relato seletivo e exigir a submissão de informações completas como condição para publicação [22,23]. Esforços coletivos de pesquisadores, de periódicos, de revisores e de agências regulatórias são necessários para promover cultura de pesquisa transparente e assegurar a prática em saúde baseada em evidências.

A maior frequência de relato seletivo de desfechos observada em periódicos com menor fator de impacto pode refletir diferenças no rigor editorial, nos processos de revisão por pares e na disponibilidade de recursos entre periódicos de diferentes níveis de prestígio [24,25]. Periódicos de alto fator de impacto tendem a adotar políticas editoriais mais rigorosas, contar com revisão por pares mais experiente e apresentar maior adesão a guias de redação, como o CONSORT [21], o que está associado a maior completude nos relatórios de ensaios clínicos [26, 27]. Já os periódicos de menor fator de impacto podem enfrentar limitações estruturais, com inclusão de recursos menores para supervisão editorial robusta, o que pode comprometer a confiabilidade e a transparência dos dados publicados [28]. Estudo meta-epidemiológico que incluiu 446 revisões sistemáticas publicadas de 2005 a 2015 constataram qualidade metodológica e de relato abaixo do ideal, mesmo em periódicos de alto impacto [29], além de frequente não conformidade com políticas de registro prospectivo de ensaios clínicos [30], o que sugeriu que desafios editoriais persistem em diferentes níveis de publicação. O fortalecimento da integridade e da transparência editorial é tarefa fundamental para aumentar a robustez dos processos editoriais, independentemente do prestígio dos periódicos [31].

A maior frequência de relato seletivo observada em ensaios clínicos de curta duração pode indicar limitações na forma como eventos adversos renais são monitorados, analisados e comunicados nesses estudos. Embora ensaios mais curtos tenham menor tempo de exposição e menor oportunidade para observar reações renais de desenvolvimento tardio ou progressivo, isso não elimina a necessidade de relato transparente dos eventos identificados durante o período de seguimento [32]. A maior frequência de relato seletivo em estudos com envolvimento de pacientes com osteoartrite merece atenção, pois essa população tende a incluir indivíduos mais velhos, frequentemente expostos ao uso prolongado de anti-inflamatórios e com maior probabilidade de comorbidades cardiovasculares, metabólicas e renais [33]. 

A maioria dos ensaios clínicos incluídos foi patrocinada pela indústria farmacêutica no período em que foi publicada, entre 1999 e 2006, época de intensa atividade comercial no setor farmacêutico, marcada por fusões e por aquisições que remodelaram o cenário da indústria [34]. Essas mudanças estruturais podem ter influenciado, ao longo do tempo, as políticas internas de transparência e a condução dos ensaios clínicos [35]. Revisão sistemática de 75 estudos observou que ensaios clínicos patrocinados pela indústria farmacêutica eram mais propensos a relatar desfechos e conclusões favoráveis à eficácia em comparação com estudos sem patrocínio da indústria [36]. Estudo observacional que analisou 822 ensaios clínicos relacionados à cirurgia da coluna também verificou que os ensaios patrocinados pela indústria apresentavam maior probabilidade de relatar resultados positivos [37]. O potencial conflito de interesses inerente aos ensaios patrocinados pela indústria reforça a necessidade de políticas públicas e editoriais que exijam a divulgação completa e transparente dos dados, bem como a implementação de estratégias para mitigar o viés de patrocínio.

O uso de limiares de incidência ou a restrição do relato apenas a eventos pré-especificados pode ocultar reações adversas que ocorrem com menor frequência, mas que são clinicamente relevantes. Tais práticas comprometem a avaliação abrangente da segurança dos medicamentos e podem expor pacientes a riscos potenciais [38]. A ausência de dados não relatados pode afetar decisões clínicas e regulatórias, dificultar a reprodutibilidade e comprometer a confiabilidade dos achados científicos [39]. O relato transparente de todos os eventos adversos, independentemente da frequência ou do tipo, é fundamental para proteger a segurança dos pacientes e manter a integridade científica.

Nossos achados têm implicações importantes para sistemas universais como o SUS, que dependem de evidências completas e transparentes para embasar decisões de incorporação, de atualização de diretrizes e de monitoramento da segurança de medicamentos [14]. O relato seletivo pode comprometer avaliações de risco-benefício, influenciar protocolos terapêuticos e dificultar a identificação precoce de eventos adversos em sistemas de farmacovigilância [40]. Práticas de maior transparência são importantes para fortalecer a segurança do paciente e apoiar políticas públicas no contexto do SUS.

Em conclusão, o relato seletivo de reações adversas renais foi frequente em ensaios clínicos com coxibs, além de potencialmente influenciado por políticas editoriais e por financiamento da pesquisa. O relato completo de todos os desfechos analisados nas pesquisas clínicas é essencial para maior transparência de eventos adversos, especialmente para avaliação crítica da segurança de medicamentos e para melhor subsidiar a tomada de decisão e a informação de políticas de saúde. Processos editoriais robustos e maior responsabilização dos atores envolvidos podem contribuir para assegurar a completude das evidências clínicas e a integridade da pesquisa científica.

## MATERIAL SUPLEMENTAR

MATERIAL SUPLEMENTAR

## Data Availability

Todo o conjunto de dados que dá suporte aos resultados deste estudo foi disponibilizado no Repositório de Dados de Pesquisa da Unicamp, disponível em https://doi.org/10.25824/redu/4DSZTA.
