# Supplementary material for: Relato seletivo de reações adversas renais relacionadas aos inibidores seletivos da ciclo-oxigenase-2 em ensaios clínicos randomizados: estudo transversal, 2023
Source: Epidemiol Serv Saude. 2026 Nov 9;35:e20260191. [Article in Portuguese] doi: 10.1590/S2237-96222026v35e20260191.pt (PMC13754238; doi:10.1590/S2237-96222026v35e20260191.pt)
Supplement: Supplementary file 1 [file 2237-9622-ress-35-e20260191-md1-en.pdf]

Supplementary Table 1. Main characteristics of the studies included in the systematic review (n=49)

Study	Location	Journal	Impact factor (2022)	Duration (months)	Clinical condition	Funder
Arber 2006 [1]	America, Europe, Africa, Asia	New England Journal of Medicine	158.5	36	Colorectal cancer	Pfizer
Bensen 1999 [2]	North America	Mayo Clinic Proceedings	8.9	1-3	Osteoarthritis	Not informed
Bertagnolli 2006 [3]	North America	New England Journal of Medicine	158.5	36	Colorectal cancer	Pfizer and National Cancer Institute
Bickham 2016 [4]	America, Europe, Africa, Asia	BMC Musculoskeletal Disorders	2.3	1-3	Rheumatoid arthritis	Merck
Bingham 2007a [5]	North America	Rheumatology	5.5	1-3	Osteoarthritis	Merck
Bingham 2007b [5]	North America	Rheumatology	5.5	1-3	Osteoarthritis	Merck
Birbara 2006 [6]	North America	Current Medical Research and Opinion	2.3	1-3	Osteoarthritis	Merck
Camu 2002 [7]	Europe	American Journal of Therapeutics	4.2	<1	Arthroplasty	Pfizer and Pharmacia Corporation
Cannon 2008 [8]	North America	Not informed	Not informed	1-3	Osteoarthritis	Merck
Chen 2016 [9]	Not informed	Pain Practice	4.2	<1	Endonasal surgery	Not informed
Coats 2004 [10]	North America	Clinical Therapeutics	3.2	1-3	Pain	Not informed
Collantes 2002 [11]	America, Europe, Africa, Asia	BMC Family Practice	2.9	1-3	Rheumatoid arthritis	Merck, Novartis, Lilly, Roche, Aventis, Pfizer, AstraZeneca, Immunex, Isotechnika, Janssen-Ortho, Knoll and Abbott
Cryer 2011a [12]	North America	Annals of Medicine	4.4	1-3	Osteoarthritis	Pozen and AstraZeneca
Cryer 2011b [12]	North America	Annals of Medicine	4.4	1-3	Osteoarthritis	Pozen and AstraZeneca
Elmets 2010 [13]	North America	JNCI-Journal of the National Cancer Institute	10.3	6-11	Actinic keratosis	Pfizer and National Cancer Institute
Essex 2018 [14]	South Korea	Journal of Pain Research	2.7	<1	Arthroplasty	Pfizer

Study	Location	Journal	Impact factor (2022)	Duration (months)	Clinical condition	Funder
Fleischmann 2006 [15]	America, Europe	Clinical Rheumatology	3.4	1-3	Osteoarthritis	Novartis
Geusens 2004 [16]	Europe, Africa, Asia, Oceania	International Journal of Clinical Practice	2.6	6-11	Rheumatoid arthritis	Novartis
Ghosh 2007 [17]	India	Journal of the Indian Medical Association	Not informed	1-3	Osteoarthritis	Not informed
Gibofsky 2003 [18]	North America	Arthritis & Rheumatism	7.379	1-3	Osteoarthritis	Pharmacia Corporation
Gottesdiener 2002 [19]	North America	Arthritis & Rheumatism	5.5	1-3	Osteoarthritis	Merck
Greenwald 2011 [20]	North America	Current Medical Research and Opinion	2.3	1-3	Rheumatoid arthritis	Merck
Grifka 2004 [21]	Germany, Canada, France, Italy	Clinical and Experimental Rheumatology	3.7	1-3	Osteoarthritis	Novartis
Hubbard 2003 [22]	Europe	British Journal of Anaesthesia	9.8	<1	Arthroplasty	Pharmacia Corporation
Ishiguro 2015 [23]	Japan	Nagoya Journal of Medical Science	0.9	<1	Pain	Astellas Pharma
Kivitz 2001 [24]	North America	Journal of International Medical Research	1.6	1-3	Osteoarthritis	Pfizer and Pharmacia Corporation
Lee 2017 [25]	South Korea	Clinics in Orthopedic Surgery	2.5	1-3	Osteoarthritis	Not informed
Lehmann 2005 [26]	Not informed	Current Medical Research and Opinion	2.3	1-3	Osteoarthritis	Novartis
Leung 2002 [27]	America, Europe, Africa, Asia, Oceania	Current Medical Research and Opinion	2.3	1-3	Osteoarthritis	Merck
Mahagna 2016 [28]	Israel	Current Medical Research and Opinion	2.6	1-3	Fibromyalgia	MSD
Matsumoto 2002 [29]	North America	Journal of Rheumatology	3.9	1-3	Rheumatoid arthritis	Merck
McKenna 2001 [30]	North America	Scandinavian Journal of Rheumatology	2.1	1-3	Osteoarthritis	Pharmacia Corporation
Pallay 2004 [31]	North America	Scandinavian Journal of Rheumatology	2.1	1-3	Pain	Merck
Papadimitrakopoulou 2008 [32]	North America	Clinical Cancer Research	11.5	1-3	Premalignant oral lesions	Pfizer
Puopolo 2007 [33]	North America	Osteoarthritis and Cartilage	7	1-3	Osteoarthritis	Merck
Rasmussen 2005 [34]	North America	Anesthesia and Analgesia	5.7	<1	Pain	Merck
Reginster 2007 [35]	United States, Canada, Australia, Europe	Annals of the Rheumatic Diseases	27.4	1-3	Osteoarthritis	Not informed

Study	Location	Journal	Impact factor (2022)	Duration (months)	Clinical condition	Funder
Schnitzer 2004 [36]	North America, Europe	Arthritis & Rheumatism	7.379	1-3	Osteoarthritis	Not informed
Schnitzer 2005 [37]	Not informed	Clinical Rheumatology	3.4	1-3	Rheumatoid arthritis	Novartis
Schnitzer 2011 [38]	Canada, United States, Germany, Italy, United Kingdom	Current Medical Research and Opinion	2.3	1-3	Osteoarthritis	Novartis
Schwartz 2002 [39]	Not informed	Clinical Pharmacology & Therapeutics	6.7	<1	Healthy older adults	Merck
Simon 1999 [40]	North America	Jama-Journal of the American Medical Association	120.7	1-3	Rheumatoid arthritis	G.D. Searle & Co.
Smugar 2006a [41]	North America	Current Medical Research and Opinion	2.3	1-3	Osteoarthritis	Merck
Smugar 2006b [41]	North America	Current Medical Research and Opinion	2.3	1-3	Osteoarthritis	Merck
Stoltz 2002 [42]	Not informed	American Journal of Gastroenterology	9.8	<1	Healthy older adults	Pharmacia Corporation
Tannenbaum 2004 [43]	Europe	Annals of the Rheumatic Diseases	27.4	1-3	Osteoarthritis	Novartis
Tsoukas 2006 [44]	North America	Blood	20.3	1-3	Hemophilic arthropathy	Merck
Wiesenhutter 2005 [45]	North America	Mayo Clinic Proceedings	8.9	1-3	Osteoarthritis	Merck
Williams 2000 [46]	North America	JCR: Journal of Clinical Rheumatology	3.4	1-3	Osteoarthritis	Not informed

Supplementary Table 2. Reasons for high risk of selective reporting of adverse renal reactions in randomized clinical trials (n=30)

Study	Reason for selective reporting
Bingham 2007a [5]	Pre-specified adverse events
Bingham 2007b [5]	Pre-specified adverse events
Camu 2002 [7]	Incidence threshold
Coats 2004 [10]	Incidence threshold
Cryer 2011a [12]	Incidence threshold
Cryer 2011b [12]	Incidence threshold
Essex 2018 [14]	Incidence threshold
Fleischmann 2006 [15]	Pre-specified adverse events
Ghosh 2007 [17]	Pre-specified adverse events
Gibofsky 2003 [18]	Incidence threshold
Grifka 2004 [21]	Pre-specified adverse events
Hubbard 2003 [22]	Incidence threshold
Ishiguro 2015 [23]	Incidence threshold
Lee 2017 [25]	Incidence threshold
Lehmann 2005 [26]	Pre-specified adverse events
Leung 2002 [27]	Pre-specified adverse events
McKenna 2001 [30]	Incidence threshold
Pallay 2004 [31]	Incidence threshold
Papadimitrakopoulou 2008 [32]	Incidence threshold
Puopolo 2007 [33]	Pre-specified adverse events
Rasmussen 2005 [34]	Incidence threshold
Reginster 2007 [35]	Incidence threshold
Schnitzer 2004 [36]	Incidence threshold
Schnitzer 2005 [37]	Incidence threshold
Schnitzer 2011 [38]	Pre-specified adverse events
Smugar 2006a [41]	Pre-specified adverse events
Smugar 2006b [41]	Pre-specified adverse events
Stoltz 2002 [42]	Incidence threshold
Tannenbaum 2004 [43]	Incidence threshold
Williams 2000 [46]	Incidence threshold

References

1. Arber N, Eagle CJ, Spicak J, Rácz I, Dite P, Hajer J, et al. Celecoxib for the prevention of colorectal adenomatous polyps. N Engl J Med. 2006;355(9): 885-95. https://doi.org10.1056/NEJMoa061652.
2. Bensen WG, Fiechtner JJ, McMillen JI, Zhao WW, Yu SS, Woods EM, et al. Treatment of osteoarthritis with celecoxib, a cyclooxygenase-2 inhibitor: a randomized controlled trial. Mayo Clin Proc. 1999;74(11): 1095-105. https://doi.org10.4065/74.11.1095.
3. Bertagnolli MM, Eagle CJ, Zauber AG, Redston M, Solomon SD, Kim K, et al. Celecoxib for the prevention of sporadic colorectal adenomas. N Engl J Med. 2006;355(9): 873-84. https://doi.org10.1056/NEJMoa061355.
4. Bickham K, Kivitz AJ, Mehta A, Frontera N, Shah S, Stryszak P, et al. Evaluation of two doses of etoricoxib, a COX-2 selective non-steroidal anti-inflammatory drug (NSAID), in the treatment of Rheumatoid Arthritis in a double-blind, randomized controlled trial. BMC Musculoskelet Disord. 2016;17: 331. https://doi.org10.1186/s12891-016-1170-0.
5. Bingham CO, 3rd, Sebba AI, Rubin BR, Ruoff GE, Kremer J, Bird S, et al. Efficacy and safety of etoricoxib 30 mg and celecoxib 200 mg in the treatment of osteoarthritis in two identically designed, randomized, placebo-controlled, non-inferiority studies. Rheumatology (Oxford). 2007;46(3): 496-507. https://doi.org10.1093/rheumatology/kel296.
6. Birbara C, Ruoff G, Sheldon E, Valenzuela C, Rodgers A, Petruschke RA, et al. Efficacy and safety of rofecoxib 12.5 mg and celecoxib 200 mg in two similarly designed osteoarthritis studies. Curr Med Res Opin. 2006;22(1): 199-210. https://doi.org10.1185/030079906X80242.
7. Camu F, Beecher T, Recker DP, Verburg KM. Valdecoxib, a COX-2-specific inhibitor, is an efficacious, opioid-sparing analgesic in patients undergoing hip arthroplasty. Am J Ther. 2002;9(1): 43-51. https://doi.org10.1097/00045391-200201000-00009.
8. Cannon CP, Chen C, Curtis SP, Viscusi J, Ahmed T, Dibattiste PM. A comparison of cardiovascular biomarkers in patients treated for three months with etoricoxib, celecoxib, ibuprofen, and placebo. Archives of Drug Information. 2008;1(1): 4-13. https://doi.org10.1111/j.1753-5174.2007.00002.x.
9. Chen H, Luo A. Safety and Efficacy Study of the Cyclooxygenase-2 Inhibitor Parecoxib Sodium Applied for Postoperative Analgesia After Endo-Nasal Operation. Pain Pract. 2016;16(4): 467-72. https://doi.org10.1111/papr.12294.
10. Coats TL, Borenstein DG, Nangia NK, Brown MT. Effects of valdecoxib in the treatment of chronic low back pain: results of a randomized, placebo-controlled trial. Clin Ther. 2004;26(8): 1249-60. https://doi.org10.1016/s0149-2918(04)80081-x.
11. Collantes E, Curtis SP, Lee KW, Casas N, McCarthy T, Melian A, et al. A multinational randomized, controlled, clinical trial of etoricoxib in the treatment of rheumatoid arthritis [ISRCTN25142273]. BMC Fam Pract. 2002;3: 10. https://doi.org10.1186/1471-2296-3-10.
12. Cryer BL, Sostek MB, Fort JG, Svensson O, Hwang C, Hochberg MC. A fixed-dose combination of naproxen and esomeprazole magnesium has comparable upper gastrointestinal tolerability to celecoxib in patients with osteoarthritis of the knee: results from two randomized, parallel-group, placebo-controlled trials. Ann Med. 2011;43(8): 594-605. https://doi.org10.3109/07853890.2011.625971.
13. Elmets CA, Viner JL, Pentland AP, Cantrell W, Lin HY, Bailey H, et al. Chemoprevention of nonmelanoma skin cancer with celecoxib: a randomized, double-blind, placebo-controlled trial. J Natl Cancer Inst. 2010;102(24): 1835-44. https://doi.org10.1093/jnci/djq442.
14. Essex MN, Choi HY, Bhadra Brown P, Cheung R. A randomized study of the efficacy and safety of parecoxib for the treatment of pain following total knee arthroplasty in Korean patients. J Pain Res. 2018;11: 427-33. https://doi.org10.2147/JPR.S147481.
15. Fleischmann R, Sheldon E, Maldonado-Cocco J, Dutta D, Yu S, Sloan VS. Lumiracoxib is effective in the treatment of osteoarthritis of the knee: a prospective randomized 13-week study versus placebo and celecoxib. Clin Rheumatol. 2006;25(1): 42-53. https://doi.org10.1007/s10067-005-1126-5.
16. Geusens P, Alten R, Rovensky J, Sloan VS, Krammer G, Kralidis G, et al. Efficacy, safety and tolerability of lumiracoxib in patients with rheumatoid arthritis. Int J Clin Pract. 2004;58(11): 1033-41. https://doi.org10.1111/j.1368-5031.2004.00398.x.
17. Ghosh S, Paul S, Das N, Bhattacharyya TK. A study on the effects of diclofenac sodium and etoricoxib in the treatment of osteoarthritis. J Indian Med Assoc. 2007;105(5): 260-2.
18. Gibofsky A, Williams GW, McKenna F, Fort JG. Comparing the efficacy of cyclooxygenase 2-specific inhibitors in treating osteoarthritis: appropriate trial design considerations and results of a randomized, placebo-controlled trial. Arthritis Rheum. 2003;48(11): 3102-11. https://doi.org10.1002/art.11330.
19. Gottesdiener K, Schnitzer T, Fisher C, Bockow B, Markenson J, Ko A, et al. Results of a randomized, dose-ranging trial of etoricoxib in patients with osteoarthritis. Rheumatology (Oxford). 2002;41(9): 1052-61. https://doi.org10.1093/rheumatology/41.9.1052.

20. Greenwald M, Peloso PM, Mandel D, Soto O, Mehta A, Frontera N, et al. Further assessment of the clinically effective dose range of etoricoxib: a randomized, double-blinded, placebo-controlled trial in rheumatoid arthritis. Curr Med Res Opin. 2011;27(10): 2033-42. https://doi.org10.1185/03007995.2011.614935.
21. Grifka JK, Zacher J, Brown JP, Seriolo B, Lee A, Moore A, et al. Efficacy and tolerability of lumiracoxib versus placebo in patients with osteoarthritis of the hand. Clin Exp Rheumatol. 2004;22(5): 589-96.
22. Hubbard RC, Naumann TM, Traylor L, Dhadda S. Parecoxib sodium has opioid-sparing effects in patients undergoing total knee arthroplasty under spinal anaesthesia. Br J Anaesth. 2003;90(2): 166-72. https://doi.org10.1093/bja/aeg038.
23. Ishiguro N, Hanaoka A, Okada T, Ito M. Efficacy and safety of celecoxib compared with placebo and etodolac for acute postoperative pain: A multicenter, double-blind, randomized, parallel-group, controlled trial. Nagoya Journal of Medical Science. 2015;77(1-2): 81-93.
24. Kivitz AJ, Moskowitz RW, Woods E, Hubbard RC, Verburg KM, Lefkowith JB, et al. Comparative efficacy and safety of celecoxib and naproxen in the treatment of osteoarthritis of the hip. J Int Med Res. 2001;29(6): 467-79. https://doi.org10.1177/147323000102900602.
25. Lee M, Yoo J, Kim JG, Kyung HS, Bin SI, Kang SB, et al. A Randomized, Multicenter, Phase III Trial to Evaluate the Efficacy and Safety of Polmacoxib Compared with Celecoxib and Placebo for Patients with Osteoarthritis. Clin Orthop Surg. 2017;9(4): 439-57. https://doi.org10.4055/cios.2017.9.4.439.
26. Lehmann R, Brzosko M, Kopsa P, Nischik R, Kreisse A, Thurston H, et al. Efficacy and tolerability of lumiracoxib 100 mg once daily in knee osteoarthritis: a 13-week, randomized, double-blind study vs. placebo and celecoxib. Curr Med Res Opin. 2005;21(4): 517-26. https://doi.org10.1185/030079905x38196.
27. Leung AT, Malmstrom K, Gallacher AE, Sarembock B, Poor G, Beaulieu A, et al. Efficacy and tolerability profile of etoricoxib in patients with osteoarthritis: A randomized, double-blind, placebo and active-comparator controlled 12-week efficacy trial. Curr Med Res Opin. 2002;18(2): 49-58. https://doi.org10.1185/030079902125000282.
28. Mahagna H, Amital D, Amital H. A randomised, double-blinded study comparing giving etoricoxib vs. placebo to female patients with fibromyalgia. Int J Clin Pract. 2016;70(2): 163-70. https://doi.org10.1111/ijcp.12760.
29. Matsumoto AK, Melian A, Mandel DR, McIlwain HH, Borenstein D, Zhao PL, et al. A randomized, controlled, clinical trial of etoricoxib in the treatment of rheumatoid arthritis. J Rheumatol. 2002;29(8): 1623-30.
30. McKenna F, Borenstein D, Wendt H, Wallemark C, Lefkowith JB, Geis GS. Celecoxib versus diclofenac in the management of osteoarthritis of the knee: a placebo-controlled, randomised, double-blind comparison. 2001;30(1): 11-18. https://doi.org10.1080/030097401750065265.
31. Pallay RM, Seger W, Adler JL, Ettlinger RE, Quaidoo EA, Lipetz R, et al. Etoricoxib reduced pain and disability and improved quality of life in patients with chronic low back pain: a 3 month, randomized, controlled trial. Scand J Rheumatol. 2004;33(4): 257-66. https://doi.org10.1080/03009740410005728.
32. Papadimitrakopoulou VA, William WN, Jr., Dannenberg AJ, Lippman SM, Lee JJ, Ondrey FG, et al. Pilot randomized phase II study of celecoxib in oral premalignant lesions. Clin Cancer Res. 2008;14(7): 2095-101. https://doi.org10.1158/1078-0432.CCR-07-4024.
33. Puopolo A, Boice JA, Fidelholtz JL, Littlejohn TW, Miranda P, Berrocal A, et al. A randomized placebo-controlled trial comparing the efficacy of etoricoxib 30 mg and ibuprofen 2400 mg for the treatment of patients with osteoarthritis. Osteoarthritis Cartilage. 2007;15(12): 1348-56. https://doi.org10.1016/j.joca.2007.05.022.
34. Rasmussen GL, Malmstrom K, Bourne MH, Jove M, Rhondeau SM, Kotey P, et al. Etoricoxib provides analgesic efficacy to patients after knee or hip replacement surgery: a randomized, double-blind, placebo-controlled study. Anesth Analg. 2005;101(4): 1104-11. https://doi.org10.1213/01.ane.0000169294.41210.9e.
35. Reginster JY, Malmstrom K, Mehta A, Bergman G, Ko AT, Curtis SP, et al. Evaluation of the efficacy and safety of etoricoxib compared with naproxen in two, 138-week randomised studies of patients with osteoarthritis. Ann Rheum Dis. 2007;66(7): 945-51. https://doi.org10.1136/ard.2006.059162.
36. Schnitzer TJ, Beier J, Geusens P, Hasler P, Patel SK, Senftleber I, et al. Efficacy and safety of four doses of lumiracoxib versus diclofenac in patients with knee or hip primary osteoarthritis: a phase II, four-week, multicenter, randomized, double-blind, placebo-controlled trial. Arthritis Rheum. 2004;51(4): 549-57. https://doi.org10.1002/art.20525.
37. Schnitzer TJ, Gitton X, Jayawardene S, Sloan VS. Lumiracoxib in the treatment of osteoarthritis, rheumatoid arthritis and acute postoperative dental pain: results of three dose-response studies. Curr Med Res Opin. 2005;21(1): 151-61. https://doi.org10.1185/030079904x20231.
38. Schnitzer TJ, Dattani ID, Seriolo B, Schneider H, Moore A, Tseng L, et al. A 13-week, multicenter, randomized, double-blind study of lumiracoxib in hip osteoarthritis. Clin Rheumatol. 2011;30(11): 1433-46. https://doi.org10.1007/s10067-011-1776-4.
39. Schwartz JI, Vandormael K, Malice MP, Kalyani RN, Lasseter KC, Holmes GB, et al. Comparison of rofecoxib, celecoxib, and naproxen on renal function in elderly subjects receiving a normal-salt diet. Clin Pharmacol Ther. 2002;72(1): 50-61. https://doi.org10.1067/mcp.2002.126182.

40. Simon LS, Weaver AL, Graham DY, Kivitz AJ, Lipsky PE, Hubbard RC, et al. Anti-inflammatory and upper gastrointestinal effects of celecoxib in rheumatoid arthritis: a randomized controlled trial. JAMA. 1999;282(20): 1921-8. https://doi.org10.1001/jama.282.20.1921.
41. Smugar SS, Schnitzer TJ, Weaver AL, Rubin BR, Polis AB, Tershakovec AM. Rofecoxib 12.5 mg, rofecoxib 25 mg, and celecoxib 200 mg in the treatment of symptomatic osteoarthritis: results of two similarly designed studies. Curr Med Res Opin. 2006;22(7): 1353-67. https://doi.org10.1185/030079906X104876.
42. Stoltz RR, Harris SI, Kuss ME, LeComte D, Talwalker S, Dhadda S, et al. Upper GI mucosal effects of parecoxib sodium in healthy elderly subjects. Am J Gastroenterol. 2002;97(1): 65-71. https://doi.org10.1111/j.1572-0241.2002.05265.x.
43. Tannenbaum H, Berenbaum F, Reginster JY, Zacher J, Robinson J, Poor G, et al. Lumiracoxib is effective in the treatment of osteoarthritis of the knee: a 13 week, randomised, double blind study versus placebo and celecoxib. Ann Rheum Dis. 2004;63(11): 1419-26. https://doi.org10.1136/ard.2003.015974.
44. Tsoukas C, Eyster ME, Shingo S, Mukhopadhyay S, Giallella KM, Curtis SP, et al. Evaluation of the efficacy and safety of etoricoxib in the treatment of hemophilic arthropathy. Blood. 2006;107(5): 1785-90. https://doi.org10.1182/blood-2004-09-3501.
45. Wiesenhutter CW, Boice JA, Ko A, Sheldon EA, Murphy FT, Wittmer BA, et al. Evaluation of the comparative efficacy of etoricoxib and ibuprofen for treatment of patients with osteoarthritis: A randomized, double-blind, placebo-controlled trial. Mayo Clin Proc. 2005;80(4): 470-9. https://doi.org10.4065/80.4.470.
46. Williams GW, Ettlinger RE, Ruderman EM, Hubbard RC, Lonien ME, Yu SS, et al. Treatment of osteoarthritis with a once-daily dosing regimen of celecoxib - A randomized, controlled trial. J Clin Rheumatol. 2000;6(2): 65-74. https://doi.org10.1097/00124743-200004000-00002.
