# Supplementary material for: Relato seletivo de reações adversas renais relacionadas aos inibidores seletivos da ciclo-oxigenase-2 em ensaios clínicos randomizados: estudo transversal, 2023
Source: Epidemiol Serv Saude. 2026 Nov 9;35:e20260191. [Article in Portuguese] doi: 10.1590/S2237-96222026v35e20260191.pt (PMC13754238; doi:10.1590/S2237-96222026v35e20260191.pt)
Supplement: Supplementary file 2 [file 2237-9622-ress-35-e20260191-md1.pdf]

**Tabela suplementar 1.** Principais características dos estudos incluídos na revisão sistemática (n=49)

| Estudo | Local | Periódico | Fator de impacto (2022) | Duração (meses) | Condição clínica | Financiador |
|---|---|---|---|---|---|---|
| Arber 2006 [1] | América, Europa, África, Ásia | New England Journal of Medicine | 158.5 | 36 | Câncer colorretal | Pfizer |
| Bensen 1999 [2] | América do Norte | Mayo Clinic Proceedings | 8.9 | 1-3 | Osteoartrite | Não informado |
| Bertagnolli 2006 [3] | América do Norte | New England Journal of Medicine | 158.5 | 36 | Câncer colorretal | Pfizer e National Cancer Institute |
| Bickham 2016 [4] | América, Europa, África, Ásia | BMC Musculoskeletal Disorders | 2.3 | 1-3 | Artrite reumatóide | Merck |
| Bingham 2007a [5] | América do Norte | Rheumatology | 5.5 | 1-3 | Osteoartrite | Merck |
| Bingham 2007b [5] | América do Norte | Rheumatology | 5.5 | 1-3 | Osteoartrite | Merck |
| Birbara 2006 [6] | América do Norte | Current Medical Research and Opinion | 2.3 | 1-3 | Osteoartrite | Merck |
| Camu 2002 [7] | Europa | American Journal of Therapeutics | 4.2 | <1 | Artroplastia | Pfizer e Pharmacia Corporation |
| Cannon 2008 [8] | América do Norte | Não informado | Não informado | 1-3 | Osteoartrite | Merck |
| Chen 2016 [9] | Não informado | Pain Practice | 4.2 | <1 | Cirurgia endonasal | Não informado |
| Coats 2004 [10] | América do Norte | Clinical Therapeutics | 3.2 | 1-3 | Dor | Não informado |
| Collantes 2002 [11] | América, Europa, África, Ásia | BMC Family Practice | 2.9 | 1-3 | Artrite reumatóide | Merck, Novartis, Lilly, Roche, Aventis, Pfizer, AstraZeneca, Immunex, Isotechnika, Janssen-Ortho, Knoll eAbbott |
| Cryer 2011a [12] | América do Norte | Annals of Medicine | 4.4 | 1-3 | Osteoartrite | Pozen e AstraZeneca |
| Cryer 2011b [12] | América do Norte | Annals of Medicine | 4.4 | 1-3 | Osteoartrite | Pozen e AstraZeneca |
| Elmets 2010 [13] | América do Norte | JNCI-Journal of the National Cancer Institute | 10.3 | 6-11 | Ceratose actínica | Pfizer aeNational Cancer Institute |
| Essex 2018 [14] | Coréia do Sul | Journal of Pain Research | 2.7 | <1 | Artroplastia | Pfizer |

| Estudo | Local | Periódico | Fator de impacto (2022) | Duração (meses) | Condição clínica | Financiador |
|---|---|---|---|---|---|---|
| Fleischmann 2006 [15] | América, Europa | Clinical Rheumatology | 3.4 | 1-3 | Osteoartrite | Novartis |
| Geusens 2004 [16] | Europa, África, Ásia, Oceania | International Journal of Clinical Practice | 2.6 | 6-11 | Artrite reumatóide | Novartis |
| Ghosh 2007 [17] | Índia | Journal of the Indian Medical Association | Não informado | 1-3 | Osteoartrite | Não informado |
| Gibofsky 2003 [18] | América do Norte | Arthritis & Rheumatism | 7.379 | 1-3 | Osteoartrite | Pharmacia Corporation |
| Gottesdiener 2002 [19] | América do Norte | Arthritis & Rheumatism | 5.5 | 1-3 | Osteoartrite | Merck |
| Greenwald 2011 [20] | América do Norte | Current Medical Research and Opinion | 2.3 | 1-3 | Artrite reumatóide | Merck |
| Grifka 2004 [21] | Alemanha, Canadá, França, Itália | Clinical and Experimental Rheumatology | 3.7 | 1-3 | Osteoartrite | Novartis |
| Hubbard 2003 [22] | Europa | British Journal of Anaesthesia | 9.8 | <1 | Artroplastia | Pharmacia Corporation |
| Ishiguro 2015 [23] | Japão | Nagoya Journal of Medical Science | 0.9 | <1 | Dor | Astellas Pharma |
| Kivitz 2001 [24] | América do Norte | Journal of International Medical Research | 1.6 | 1-3 | Osteoartrite | Pfizer e Pharmacia Corporation |
| Lee 2017 [25] | Coréia do Sul | Clinics in Orthopedic Surgery | 2.5 | 1-3 | Osteoartrite | Não informado |
| Lehmann 2005 [26] | Não informado | Current Medical Research and Opinion | 2.3 | 1-3 | Osteoartrite | Novartis |
| Leung 2002 [27] | América, Europa, África, Ásia, Oceania | Current Medical Research and Opinion | 2.3 | 1-3 | Osteoartrite | Merck |
| Mahagna 2016 [28] | Israel | Current Medical Research and Opinion | 2.6 | 1-3 | Fibromialgia | MSD |
| Matsumoto 2002 [29] | América do Norte | Journal of Rheumatology | 3.9 | 1-3 | Artrite reumatóide | Merck |
| McKenna 2001 [30] | América do Norte | Scandinavian Journal of Rheumatology | 2.1 | 1-3 | Osteoartrite | Pharmacia Corporation |
| Pallay 2004 [31] | América do Norte | Scandinavian Journal of Rheumatology | 2.1 | 1-3 | Dor | Merck |
| Papadimitrakopoulou 2008 [32] | América do Norte | Clinical Cancer Research | 11.5 | 1-3 | Lesões pré-malignas orais | Pfizer |
| Puopolo 2007 [33] | América do Norte | Osteoarthritis and Cartilage | 7 | 1-3 | Osteoartrite | Merck |
| Rasmussen 2005 [34] | América do Norte | Anesthesia and Analgesia | 5.7 | <1 | Dor | Merck |
| Reginster 2007 [35] | Estados Unidos, Canadá, | Annals of the Rheumatic Diseases | 27.4 | 1-3 | Osteoartrite | Não informado |

| Estudo | Local | Periódico | Fator de impacto (2022) | Duração (meses) | Condição clínica | Financiador |
|---|---|---|---|---|---|---|
| | Austrália, Europa | | | | | |
| Schnitzer 2004 [36] | América do Norte, Europa | Arthritis & Rheumatism | 7.379 | 1-3 | Osteoartrite | Não informado |
| Schnitzer 2005 [37] | Não informado | Clinical Rheumatology | 3.4 | 1-3 | Artrite reumatóide | Novartis |
| Schnitzer 2011 [38] | Canadá, Estados Unidos, Alemanha, Itália, Reino Unido | Current Medical Research and Opinion | 2.3 | 1-3 | Osteoartrite | Novartis |
| Schwartz 2002 [39] | Não informado | Clinical Pharmacology & Therapeutics | 6.7 | <1 | Idosos saudáveis | Merck |
| Simon 1999 [40] | América do Norte | Jama-Journal of the American Medical Association | 120.7 | 1-3 | Artrite reumatóide | G.D. Searle & Co |
| Smugar 2006a [41] | América do Norte | Current Medical Research and Opinion | 2.3 | 1-3 | Osteoartrite | Merck |
| Smugar 2006b [41] | América do Norte | Current Medical Research and Opinion | 2.3 | 1-3 | Osteoartrite | Merck |
| Stoltz 2002 [42] | Não informado | American Journal of Gastroenterology | 9.8 | <1 | Idosos saudáveis | Pharmacia Corporation |
| Tannenbaum 2004 [43] | Europa | Annals of the Rheumatic Diseases | 27.4 | 1-3 | Osteoartrite | Novartis |
| Tsoukas 2006 [44] | América do Norte | Blood | 20.3 | 1-3 | Artropatia hemofílica | Merck |
| Wiesenhutter 2005 [45] | América do Norte | Mayo Clinic Proceedings | 8.9 | 1-3 | Osteoartrite | Merck |
| Williams 2000 [46] | América do Norte | JCR: Journal of Clinical Rheumatology | 3.4 | 1-3 | Osteoartrite | Não informado |

**Tabela suplementar 2.** Motivos de alto risco de relato seletivo de reações adversas renais nos ensaios clínicos randomizados (n=30)

| **Estudo** | **Motivo de relato seletivo** |
|---|---|
| Bingham 2007a [5] | Eventos adversos pré-especificados |
| Bingham 2007b [5] | Eventos adversos pré-especificados |
| Camu 2002 [7] | Limiar de incidência |
| Coats 2004 [10] | Limiar de incidência |
| Cryer 2011a [12] | Limiar de incidência |
| Cryer 2011b [12] | Limiar de incidência |
| Essex 2018 [14] | Limiar de incidência |
| Fleischmann 2006 [15] | Eventos adversos pré-especificados |
| Ghosh 2007 [17] | Eventos adversos pré-especificados |
| Gibofsky 2003 [18] | Limiar de incidência |
| Grifka 2004 [21] | Eventos adversos pré-especificados |
| Hubbard 2003 [22] | Limiar de incidência |
| Ishiguro 2015 [23] | Limiar de incidência |
| Lee 2017 [25] | Limiar de incidência |
| Lehmann 2005 [26] | Eventos adversos pré-especificados |
| Leung 2002 [27] | Eventos adversos pré-especificados |
| McKenna 2001 [30] | Limiar de incidência |
| Pallay 2004 [31] | Limiar de incidência |
| Papadimitrakopoulou 2008 [32] | Limiar de incidência |
| Puopolo 2007 [33] | Eventos adversos pré-especificados |
| Rasmussen 2005 [34] | Limiar de incidência |
| Reginster 2007 [35] | Limiar de incidência |
| Schnitzer 2004 [36] | Limiar de incidência |
| Schnitzer 2005 [37] | Limiar de incidência |
| Schnitzer 2011 [38] | Eventos adversos pré-especificados |
| Smugar 2006a [41] | Eventos adversos pré-especificados |
| Smugar 2006b [41] | Eventos adversos pré-especificados |
| Stoltz 2002 [42] | Limiar de incidência |
| Tannenbaum 2004 [43] | Limiar de incidência |
| Williams 2000 [46] | Limiar de incidência |

## Referências


1. Arber N, Eagle CJ, Spicak J, Rácz I, Dite P, Hajer J, et al. Celecoxib for the prevention of colorectal adenomatous polyps. N Engl J Med. 2006;355(9): 885-95. https://doi.org10.1056/NEJMoa061652.
2. Bensen WG, Fiechtner JJ, McMillen JI, Zhao WW, Yu SS, Woods EM, et al. Treatment of osteoarthritis with celecoxib, a cyclooxygenase-2 inhibitor: a randomized controlled trial. Mayo Clin Proc. 1999;74(11): 1095-105. https://doi.org10.4065/74.11.1095.
3. Bertagnolli MM, Eagle CJ, Zauber AG, Redston M, Solomon SD, Kim K, et al. Celecoxib for the prevention of sporadic colorectal adenomas. N Engl J Med. 2006;355(9): 873-84. https://doi.org10.1056/NEJMoa061355.
4. Bickham K, Kivitz AJ, Mehta A, Frontera N, Shah S, Stryszak P, et al. Evaluation of two doses of etoricoxib, a COX-2 selective non-steroidal anti-inflammatory drug (NSAID), in the treatment of Rheumatoid Arthritis in a double-blind, randomized controlled trial. BMC Musculoskelet Disord. 2016;17: 331. https://doi.org10.1186/s12891-016-1170-0.
5. Bingham CO, 3rd, Sebba AI, Rubin BR, Ruoff GE, Kremer J, Bird S, et al. Efficacy and safety of etoricoxib 30 mg and celecoxib 200 mg in the treatment of osteoarthritis in two identically designed, randomized, placebo-controlled, non-inferiority studies. Rheumatology (Oxford). 2007;46(3): 496-507. https://doi.org10.1093/rheumatology/kel296.
6. Birbara C, Ruoff G, Sheldon E, Valenzuela C, Rodgers A, Petruschke RA, et al. Efficacy and safety of rofecoxib 12.5 mg and celecoxib 200 mg in two similarly designed osteoarthritis studies. Curr Med Res Opin. 2006;22(1): 199-210. https://doi.org10.1185/030079906X80242.
7. Camu F, Beecher T, Recker DP, Verburg KM. Valdecoxib, a COX-2-specific inhibitor, is an efficacious, opioid-sparing analgesic in patients undergoing hip arthroplasty. Am J Ther. 2002;9(1): 43-51. https://doi.org10.1097/00045391-200201000-00009.
8. Cannon CP, Chen C, Curtis SP, Viscusi J, Ahmed T, Dibattiste PM. A comparison of cardiovascular biomarkers in patients treated for three months with etoricoxib, celecoxib, ibuprofen, and placebo. Archives of Drug Information. 2008;1(1): 4-13. https://doi.org10.1111/j.1753-5174.2007.00002.x.
9. Chen H, Luo A. Safety and Efficacy Study of the Cyclooxygenase-2 Inhibitor Parecoxib Sodium Applied for Postoperative Analgesia After Endo-Nasal Operation. Pain Pract. 2016;16(4): 467-72. https://doi.org10.1111/papr.12294.
10. Coats TL, Borenstein DG, Nangia NK, Brown MT. Effects of valdecoxib in the treatment of chronic low back pain: results of a randomized, placebo-controlled trial. Clin Ther. 2004;26(8): 1249-60. https://doi.org10.1016/s0149-2918(04)80081-x.
11. Collantes E, Curtis SP, Lee KW, Casas N, McCarthy T, Melian A, et al. A multinational randomized, controlled, clinical trial of etoricoxib in the treatment of rheumatoid arthritis [ISRCTN25142273]. BMC Fam Pract. 2002;3: 10. https://doi.org10.1186/1471-2296-3-10.
12. Cryer BL, Sostek MB, Fort JG, Svensson O, Hwang C, Hochberg MC. A fixed-dose combination of naproxen and esomeprazole magnesium has comparable upper gastrointestinal tolerability to celecoxib in patients with osteoarthritis of the knee: results from two randomized, parallel-group, placebo-controlled trials. Ann Med. 2011;43(8): 594-605. https://doi.org10.3109/07853890.2011.625971.
13. Elmets CA, Viner JL, Pentland AP, Cantrell W, Lin HY, Bailey H, et al. Chemoprevention of nonmelanoma skin cancer with celecoxib: a randomized, double-blind, placebo-controlled trial. J Natl Cancer Inst. 2010;102(24): 1835-44. https://doi.org10.1093/jnci/djq442.
14. Essex MN, Choi HY, Bhadra Brown P, Cheung R. A randomized study of the efficacy and safety of parecoxib for the treatment of pain following total knee arthroplasty in Korean patients. J Pain Res. 2018;11: 427-33. https://doi.org10.2147/JPR.S147481.
15. Fleischmann R, Sheldon E, Maldonado-Cocco J, Dutta D, Yu S, Sloan VS. Lumiracoxib is effective in the treatment of osteoarthritis of the knee: a prospective randomized 13-week study versus placebo and celecoxib. Clin Rheumatol. 2006;25(1): 42-53. https://doi.org10.1007/s10067-005-1126-5.
16. Geusens P, Alten R, Rovensky J, Sloan VS, Krammer G, Kralidis G, et al. Efficacy, safety and tolerability of lumiracoxib in patients with rheumatoid arthritis. Int J Clin Pract. 2004;58(11): 1033-41. https://doi.org10.1111/j.1368-5031.2004.00398.x.
17. Ghosh S, Paul S, Das N, Bhattacharyya TK. A study on the effects of diclofenac sodium and etoricoxib in the treatment of osteoarthritis. J Indian Med Assoc. 2007;105(5): 260-2.
18. Gibofsky A, Williams GW, McKenna F, Fort JG. Comparing the efficacy of cyclooxygenase 2-specific inhibitors in treating osteoarthritis: appropriate trial design considerations and results of a randomized, placebo-controlled trial. Arthritis Rheum. 2003;48(11): 3102-11. https://doi.org10.1002/art.11330.
19. Gottesdiener K, Schnitzer T, Fisher C, Bockow B, Markenson J, Ko A, et al. Results of a randomized, dose-ranging trial of etoricoxib in patients with osteoarthritis. Rheumatology (Oxford). 2002;41(9): 1052-61. https://doi.org10.1093/rheumatology/41.9.1052.

20. Greenwald M, Peloso PM, Mandel D, Soto O, Mehta A, Frontera N, et al. Further assessment of the clinically effective dose range of etoricoxib: a randomized, double-blinded, placebo-controlled trial in rheumatoid arthritis. Curr Med Res Opin. 2011;27(10): 2033-42. https://doi.org10.1185/03007995.2011.614935.
21. Grifka JK, Zacher J, Brown JP, Seriolo B, Lee A, Moore A, et al. Efficacy and tolerability of lumiracoxib versus placebo in patients with osteoarthritis of the hand. Clin Exp Rheumatol. 2004;22(5): 589-96.
22. Hubbard RC, Naumann TM, Traylor L, Dhadda S. Parecoxib sodium has opioid-sparing effects in patients undergoing total knee arthroplasty under spinal anaesthesia. Br J Anaesth. 2003;90(2): 166-72. https://doi.org10.1093/bja/aeg038.
23. Ishiguro N, Hanaoka A, Okada T, Ito M. Efficacy and safety of celecoxib compared with placebo and etodolac for acute postoperative pain: A multicenter, double-blind, randomized, parallel-group, controlled trial. Nagoya Journal of Medical Science. 2015;77(1-2): 81-93.
24. Kivitz AJ, Moskowitz RW, Woods E, Hubbard RC, Verburg KM, Lefkowith JB, et al. Comparative efficacy and safety of celecoxib and naproxen in the treatment of osteoarthritis of the hip. J Int Med Res. 2001;29(6): 467-79. https://doi.org10.1177/147323000102900602.
25. Lee M, Yoo J, Kim JG, Kyung HS, Bin SI, Kang SB, et al. A Randomized, Multicenter, Phase III Trial to Evaluate the Efficacy and Safety of Polmacoxib Compared with Celecoxib and Placebo for Patients with Osteoarthritis. Clin Orthop Surg. 2017;9(4): 439-57. https://doi.org10.4055/cios.2017.9.4.439.
26. Lehmann R, Brzosko M, Kopsa P, Nischik R, Kreisse A, Thurston H, et al. Efficacy and tolerability of lumiracoxib 100 mg once daily in knee osteoarthritis: a 13-week, randomized, double-blind study vs. placebo and celecoxib. Curr Med Res Opin. 2005;21(4): 517-26. https://doi.org10.1185/030079905x38196.
27. Leung AT, Malmstrom K, Gallacher AE, Sarembock B, Poor G, Beaulieu A, et al. Efficacy and tolerability profile of etoricoxib in patients with osteoarthritis: A randomized, double-blind, placebo and active-comparator controlled 12-week efficacy trial. Curr Med Res Opin. 2002;18(2): 49-58. https://doi.org10.1185/030079902125000282.
28. Mahagna H, Amital D, Amital H. A randomised, double-blinded study comparing giving etoricoxib vs. placebo to female patients with fibromyalgia. Int J Clin Pract. 2016;70(2): 163-70. https://doi.org10.1111/ijcp.12760.
29. Matsumoto AK, Melian A, Mandel DR, McIlwain HH, Borenstein D, Zhao PL, et al. A randomized, controlled, clinical trial of etoricoxib in the treatment of rheumatoid arthritis. J Rheumatol. 2002;29(8): 1623-30.
30. McKenna F, Borenstein D, Wendt H, Wallemark C, Lefkowith JB, Geis GS. Celecoxib versus diclofenac in the management of osteoarthritis of the knee: a placebo-controlled, randomised, double-blind comparison. 2001;30(1): 11-18. https://doi.org10.1080/030097401750065265.
31. Pallay RM, Seger W, Adler JL, Ettlinger RE, Quaidoo EA, Lipetz R, et al. Etoricoxib reduced pain and disability and improved quality of life in patients with chronic low back pain: a 3 month, randomized, controlled trial. Scand J Rheumatol. 2004;33(4): 257-66. https://doi.org10.1080/03009740410005728.
32. Papadimitrakopoulou VA, William WN, Jr., Dannenberg AJ, Lippman SM, Lee JJ, Ondrey FG, et al. Pilot randomized phase II study of celecoxib in oral premalignant lesions. Clin Cancer Res. 2008;14(7): 2095-101. https://doi.org10.1158/1078-0432.CCR-07-4024.
33. Puopolo A, Boice JA, Fidelholtz JL, Littlejohn TW, Miranda P, Berrocal A, et al. A randomized placebo-controlled trial comparing the efficacy of etoricoxib 30 mg and ibuprofen 2400 mg for the treatment of patients with osteoarthritis. Osteoarthritis Cartilage. 2007;15(12): 1348-56. https://doi.org10.1016/j.joca.2007.05.022.
34. Rasmussen GL, Malmstrom K, Bourne MH, Jove M, Rhondeau SM, Kotey P, et al. Etoricoxib provides analgesic efficacy to patients after knee or hip replacement surgery: a randomized, double-blind, placebo-controlled study. Anesth Analg. 2005;101(4): 1104-11. https://doi.org10.1213/01.ane.0000169294.41210.9e.
35. Reginster JY, Malmstrom K, Mehta A, Bergman G, Ko AT, Curtis SP, et al. Evaluation of the efficacy and safety of etoricoxib compared with naproxen in two, 138-week randomised studies of patients with osteoarthritis. Ann Rheum Dis. 2007;66(7): 945-51. https://doi.org10.1136/ard.2006.059162.
36. Schnitzer TJ, Beier J, Geusens P, Hasler P, Patel SK, Senftleber I, et al. Efficacy and safety of four doses of lumiracoxib versus diclofenac in patients with knee or hip primary osteoarthritis: a phase II, four-week, multicenter, randomized, double-blind, placebo-controlled trial. Arthritis Rheum. 2004;51(4): 549-57. https://doi.org10.1002/art.20525.
37. Schnitzer TJ, Gitton X, Jayawardene S, Sloan VS. Lumiracoxib in the treatment of osteoarthritis, rheumatoid arthritis and acute postoperative dental pain: results of three dose-response studies. Curr Med Res Opin. 2005;21(1): 151-61. https://doi.org10.1185/030079904x20231.
38. Schnitzer TJ, Dattani ID, Seriolo B, Schneider H, Moore A, Tseng L, et al. A 13-week, multicenter, randomized, double-blind study of lumiracoxib in hip osteoarthritis. Clin Rheumatol. 2011;30(11): 1433-46. https://doi.org10.1007/s10067-011-1776-4.
39. Schwartz JI, Vandormael K, Malice MP, Kalyani RN, Lasseter KC, Holmes GB, et al. Comparison of rofecoxib, celecoxib, and naproxen on renal function in elderly subjects receiving a normal-salt diet. Clin Pharmacol Ther. 2002;72(1): 50-61. https://doi.org10.1067/mcp.2002.126182.

40. Simon LS, Weaver AL, Graham DY, Kivitz AJ, Lipsky PE, Hubbard RC, et al. Anti-inflammatory and upper gastrointestinal effects of celecoxib in rheumatoid arthritis: a randomized controlled trial. JAMA. 1999;282(20): 1921-8. https://doi.org10.1001/jama.282.20.1921.
41. Smugar SS, Schnitzer TJ, Weaver AL, Rubin BR, Polis AB, Tershakovec AM. Rofecoxib 12.5 mg, rofecoxib 25 mg, and celecoxib 200 mg in the treatment of symptomatic osteoarthritis: results of two similarly designed studies. Curr Med Res Opin. 2006;22(7): 1353-67. https://doi.org10.1185/030079906X104876.
42. Stoltz RR, Harris SI, Kuss ME, LeComte D, Talwalker S, Dhadda S, et al. Upper GI mucosal effects of parecoxib sodium in healthy elderly subjects. Am J Gastroenterol. 2002;97(1): 65-71. https://doi.org10.1111/j.1572-0241.2002.05265.x.
43. Tannenbaum H, Berenbaum F, Reginster JY, Zacher J, Robinson J, Poor G, et al. Lumiracoxib is effective in the treatment of osteoarthritis of the knee: a 13 week, randomised, double blind study versus placebo and celecoxib. Ann Rheum Dis. 2004;63(11): 1419-26. https://doi.org10.1136/ard.2003.015974.
44. Tsoukas C, Eyster ME, Shingo S, Mukhopadhyay S, Giallella KM, Curtis SP, et al. Evaluation of the efficacy and safety of etoricoxib in the treatment of hemophilic arthropathy. Blood. 2006;107(5): 1785-90. https://doi.org10.1182/blood-2004-09-3501.
45. Wiesenhutter CW, Boice JA, Ko A, Sheldon EA, Murphy FT, Wittmer BA, et al. Evaluation of the comparative efficacy of etoricoxib and ibuprofen for treatment of patients with osteoarthritis: A randomized, double-blind, placebo-controlled trial. Mayo Clin Proc. 2005;80(4): 470-9. https://doi.org10.4065/80.4.470.
46. Williams GW, Ettlinger RE, Ruderman EM, Hubbard RC, Lonien ME, Yu SS, et al. Treatment of osteoarthritis with a once-daily dosing regimen of celecoxib - A randomized, controlled trial. J Clin Rheumatol. 2000;6(2): 65-74. https://doi.org10.1097/00124743-200004000-00002.
