# Supplementary material for: Relato seletivo de reações adversas renais relacionadas aos inibidores seletivos da ciclo-oxigenase-2 em ensaios clínicos randomizados: estudo transversal, 2023
Source: Epidemiol Serv Saude. 2026 Nov 9;35:e20260191. [Article in Portuguese] doi: 10.1590/S2237-96222026v35e20260191.pt (PMC13754238; doi:10.1590/S2237-96222026v35e20260191.pt)
Supplement: Translation

# Translation: Selective reporting of adverse renal reactions associated with selective cyclooxygenase-2 inhibitors in randomized clinical trials: a cross-sectional study, 2023

**DOI:** 10.1590/S2237-96222026v35e20260191.en

**Authors:** Tayanny Margarida Menezes Almeida Biase, Marcus Tolentino Silva, Tais Freire Galvão

**Affiliations:** 1 Universidade Estadual de Campinas, Faculdade de Ciências Farmacêuticas, Campinas, SP, Brazil; 2 Universidade de Brasília, Departamento de Saúde Coletiva, Brasília, DF, Brazil

**Keywords:** Publication Bias, Peer Review, Adverse Event, Cyclooxygenase 2 Inhibitors, Cross-Sectional Studies

## Abstract

**Objective:**

To identify the frequency of selective outcome reporting in randomized clinical trials evaluating the renal safety of selective cyclooxygenase-2 inhibitors (coxibs) and to explore factors associated with its occurrence.

**Methods:**

This is a cross-sectional analysis based on a previous systematic review regarding the renal effects of coxibs. Selective outcome reporting was defined as cases in which studies presented results only for pre-defined adverse events or applied an incidence threshold for reporting renal reactions. Data were collected on impact factor, peer-review duration, study location, type of coxib and funding sources. Continuous variables were described using means and standard deviations, while categorical variables were described using frequencies. Factors associated with selective reporting were tested using the chi-square test or t-test (p-value<0.05).

**Results:**

Of the 49 studies included, 30 showed selective reporting; 19 established thresholds for reporting reactions, and 11 reported only pre-specified events. Omission of adverse reactions was more frequent in journals with lower impact factors (n=29; 6.1±6.5). Peer review duration was longer in trials without reporting bias (n=11) than in those with the bias (n=15), though not significantly (p-value 0.470). Studies on osteoarthritis showed a higher frequency of selective reporting (20/26 versus 10/23; p-value 0.016), and trials lasting ≤6 weeks showed a higher proportion of the bias (17/22) compared to those of longer duration (13/27; p-value 0.037). The majority of studies (41/49) were industry-funded.

**Conclusions:**

Selective reporting of adverse renal reactions was common in coxib clinical trials, potentially influenced by editorial policies and research sponsorship.

## Introduction

Selective cyclooxygenase-2 inhibitor anti-inflammatory drugs (coxibs) were introduced to the market in the 1990s with the promise of reducing gastrointestinal toxicity compared to traditional non-steroidal anti-inflammatory drugs [1]. Shortly after their widespread use, concerns regarding cardiovascular safety emerged [2], leading to the 2004 market withdrawal of rofecoxib, the first coxib to be discontinued for this reason [3,4]. This process became emblematic of issues surrounding transparency in adverse event reporting. In the case of a randomized study comparing rofecoxib and naproxen in patients with rheumatoid arthritis [5], the original publication did not fully present relevant cardiovascular data [6]. Subsequently, an expression of concern was published, noting that three additional cases of myocardial infarction, all in the rofecoxib group, had not been included in the data submitted to the journal; this underscored the importance of complete adverse event reporting in clinical trials [6].

These safety concerns led to regulatory restrictions, risk communication strategies and prescribing guidelines aimed at limiting inappropriate use [7]. Despite these measures intended to promote rational use, use of coxibs has continued to rise in Brazil [8] and in various settings, particularly among populations with multimorbidity and chronic pain [9].

A systematic review of clinical trials on coxibs found that these drugs increase the risk of adverse renal effects, including hypertension and edema, with moderate to low certainty of evidence [10]. Selective reporting of these adverse events was common, contributing to risk of bias and imprecision in the results [10]. Omission of adverse drug reactions in publications can mislead physicians and policymakers, leading to underestimation of potential harms and a false sense of security, which may result in greater patient exposure to serious harm [11]. Selective outcome reporting can also directly affect clinical decision-making and public health policies, particularly regarding drug safety assessments [12,13]. This practice has direct implications for universal health systems, such as the Brazilian Unified Health System (*Sistema* Único *de Saúde*, SUS), which relies on robust and comprehensive evidence for decisions regarding adoption of new technologies, updating of clinical protocols and post-marketing safety monitoring [14].

Identifying factors associated with reporting bias can contribute to the debate on the transparency and reliability of clinical evidence for healthcare decision-making. In this context, this study aimed to identify the frequency of selective reporting of adverse renal reactions and to explore study characteristics associated with the occurrence of this outcome in clinical trials included in a systematic review that assessed the renal effects of coxibs.

## Methods

### Study design and participants

This is a cross-sectional analysis based on a previous systematic review that assessed adverse renal effects of coxibs [10]. The randomized clinical trials included in the systematic review were eligible for this analysis [10].

### Setting

The systematic review followed a protocol registered with the International Prospective Register of Systematic Reviews (CRD42022380227). The review was based on randomized clinical trials evaluating the renal effects of coxibs (celecoxib, etoricoxib, lumiracoxib, parecoxib and valdecoxib) compared with placebo. Database searches were conducted up to March 2024, using a peer-reviewed search strategy. Two independent reviewers performed study selection, data extraction and risk-of-bias assessment. Meta-analysis, meta-regression and assessment of the certainty of the evidence were also conducted [10].

### Variables, data sources and measurement

The dependent variable in this study was selective outcome reporting, dichotomized as present or absent, defined as the omission, incomplete presentation or insufficient description of adverse renal reactions that had been previously assessed or were expected in the context of the clinical trials, thereby hindering interpretation of drug safety profiles. Selective outcome reporting was classified as present in studies at high risk of bias regarding item No. 6 of the Cochrane Risk of Bias tool [15]. Studies were classified as having high risk of bias in this domain if they: (i) adopted an incidence threshold for reporting adverse events (i.e., defined a minimum event frequency for reporting adverse reactions in the final publication) or (ii) reported only pre-specified adverse events, omitting other adverse events that occurred during the trial but were not on the initial list. Studies that did not meet these criteria were classified as not having selective reporting.

The following information was collected: journal name, publication date, study location, duration (in weeks), type of coxib, participant clinical condition, number of participants, and the pharmaceutical company manufacturing the coxibs at the time the studies were published. Submission and acceptance dates were obtained from the full-text articles or journal websites to calculate peer-review duration (in days). For clarity, we defined the time from submission to acceptance as being the peer-review duration. The 2022 Journal Impact Factor and the gold open access status of the journals in which the studies were published were obtained from the Clarivate Journal Citation Reports in September 2023.

The research data are available in full from the Unicamp Research Data Repository [16]. 

### Statistical methods

Descriptive statistics were calculated, including frequencies, means and standard deviations (SD). Factors associated with selective reporting were assessed using Pearson’s chi-square test for categorical variables and Student’s t-test for continuous variables, whereby a p-value<0.05 was taken to be significant. All analyses were performed using Stata software, version 14.2 (College Station, TX).

## Results

Selective reporting was observed in 30 of the 49 studies included in the systematic review. The studies were conducted in North America (n=35) and Europe (n=14). The majority were published in journals with an impact factor (n=47); 31 studies were published between 1999 and 2006; 14 appeared in rheumatology journals and 10 in general medicine journals (Table 1 and Supplementary Table 1).

The primary reason for selective reporting was the use of incidence thresholds to report adverse reactions (n=19), with minimum frequency cutoffs of 1-2% (n=4), 3% (n=8) or 4-5% (n=7) established for reporting occurrence of adverse reactions. Eleven studies reported only pre-specified adverse events (Supplementary Table 2).

It was possible to calculate the editorial processing time for 25 studies. Studies without reporting bias (n=11) had longer peer-review duration, though not significantly so, (190.7±155.3 days) than those with reporting bias (n=15; 155.1±91.4 days; p-value 0.470). The number of participants was also higher in studies without reporting bias (n=19; 671.7±540.7) compared to trials with bias (n=30; 527.2±452.6; p-value 0.317). Selective reporting was significantly more frequent in journals with lower impact factors (n=29; 6.1±6.5) compared to those with higher prestige (n=18; 29.0±54.5; p-value 0.029). No significant differences were observed regarding open access, publication date or type of coxib (p-value>0.05) (Table 2).

**Table 1:** Frequency of characteristics of clinical trials involving selective cyclooxygenase-2 inhibitors (coxibs) according to occurrence of selective reporting of adverse renal reactions (n=49)

Variable	With selective reporting (n=30)	Without selective reporting (n=19)	p-value^a^
Publication date			0.551
1999-2006	18	13	
2007-2018	12	6	
Study location			0.354
North America	20	15	
Europe	10	4	
Coxib type			
Celecoxib	16	9	0.684
Etoricoxib	8	9	0.138
Lumiracoxib	7	1	0.095
Parecoxib	3	1	0.555
Valdecoxib	2	0	0.250
Clinical condition			0.016
Osteoarthritis	20	6	
Other	10	13	
Study duration (weeks)			0.037
<6	17	5	
>6	13	14	
Pharmaceutical company			
Merck	22	8	0.029
Pfizer	23	14	0.813
Novartis	24	17	0.382

a Pearson’s chi-square test.

**Table 2:** Mean and standard deviation (SD) of clinical trial characteristics according to occurrence of selective reporting of adverse renal reactions (n=49)

Variable	With selective reporting		Without selective reporting		p-value^a^
	n	Mean±SD	n	Mean±SD	
Impact factor	29	6.1±6.5	18	29.0±54.5	0.029
Open access percentage	27	33.1±28.9	18	36.1±34.5	0.773
Number of participants	30	527.2±452.6	19	671.7±540.7	0.317
Peer-review duration (days)	15	155.1±91.4	11	190.7±155.3	0.470

a Student’s t-test

Studies related to osteoarthritis showed higher frequency of selective reporting (20/26 versus 10/23; p-value 0.016). Similarly, trials lasting up to six weeks showed a higher proportion of selective reporting (17/22) compared to those of longer duration (13/27; p-value 0.037).

The majority of the studies (41/49) received funding from pharmaceutical companies manufacturing coxibs at the time of publication. The main manufacturers funding the trials were Merck (n=19), Pfizer (n=12) and Novartis (n=8). Studies funded by Merck showed a lower frequency of selective reporting (8/19 vs. 22/30; p-value 0.029) (Table 1).

## Discussion

Selective reporting of renal reactions was high in randomized clinical trials evaluating coxibs. Reporting bias was more common in studies published in journals with lower impact factors, in short-term trials and in research involving patients with osteoarthritis. Pharmaceutical company funding was predominant among the studies included, with variations between sponsors regarding occurrence of selective reporting.

This analysis was based on a previously published systematic review [10], the results of which were directly influenced by its eligibility criteria, the selection and data extraction processes conducted by independent reviewers, and the risk-of-bias assessment. Potential methodological limitations of the original review may impact the present analysis. The time elapsed between article submission and acceptance was taken to be the peer-review duration, rather than a measure of peer-review quality. This variable may be influenced by factors beyond the authors’ control, such as internal administrative steps, the time taken by authors to respond to review requests, the number of review rounds, reviewer availability and journal-specific editorial practices. Factors such as the regulatory context at the time of publication, the potential influence of peer reviewers and editors, and specific journal editorial practices were not assessed. These aspects can play a significant role in the occurrence of selective reporting, and their omission from this analysis may have limited the interpretation of the findings [17,18].

The high frequency of selective outcome reporting observed in this study is consistent with findings from other areas of health research. A 2024 study analyzing clinical trials on antibiotic use in third-molar surgeries identified selective outcome reporting in 88% of the studies when comparing registered protocols with published articles [19]. Similarly, a systematic review of clinical trials published in hematology journals identified selective outcome reporting in a large proportion of the trials, with discrepancies primarily related to omission, addition or alteration of primary outcomes [20].

The results of this study highlighted the need for structural improvements to foster greater transparency and reliability in scientific communication. Several measures are recommended in order to address these challenges and enhance transparency in science, including strengthening requirements for prospective clinical trial registration, ensuring the full publication of adverse event data regardless of observed frequency, and adhering to scientific reporting guidelines such as the Consolidated Standards of Reporting Trials (CONSORT) [21]. Improving the peer-review process is also crucial, involving better training for reviewers and editors to detect selective reporting practices and to mandate the submission of complete information as a condition for publication [22,23]. Collective efforts by researchers, journals, reviewers and regulatory agencies are required to promote a culture of transparent research and ensure evidence-based healthcare practice.

The higher frequency of selective outcome reporting observed in journals with lower impact factors may reflect differences in editorial rigor, peer-review processes and resource availability among journals of varying prestige [24,25]. High-impact-factor journals tend to adopt stricter editorial policies, rely on more experienced peer reviewers and demonstrate greater adherence to reporting guidelines, such as CONSORT [21], which is associated with more complete clinical trial reporting [26, 27]. Conversely, journals with lower impact factors may face structural limitations, including fewer resources for robust editorial oversight, which can compromise the reliability and transparency of published data [28]. A meta-epidemiological study involving 446 systematic reviews published between 2005 and 2015 found suboptimal methodological and reporting quality, even in high-impact journals [29], as well as frequent non-compliance with policies regarding prospective clinical trial registration [30], suggesting that editorial challenges persist across different levels of publication. Strengthening editorial integrity and transparency is essential to enhancing the robustness of editorial processes, regardless of a journal’s prestige [31].

The higher frequency of selective reporting observed in short-term clinical trials may indicate limitations in how adverse renal events are monitored, analyzed and reported in these studies. Although shorter trials involve shorter exposure times and fewer opportunities to observe renal reactions with a delayed or progressive onset, this does not eliminate the need for transparent reporting of events identified during the follow-up period [32]. The higher frequency of selective reporting in studies involving patients with osteoarthritis warrants attention, as this population tends to include older individuals who are frequently exposed to long-term anti-inflammatory use and have a higher likelihood of cardiovascular, metabolic and renal comorbidities [33].

The majority of the clinical trials included were sponsored by the pharmaceutical industry during the period of their publication (1999-2006), a time of intense commercial activity in the sector, marked by mergers and acquisitions that reshaped the industry landscape [34]. These structural changes may have influenced internal transparency policies and the conduct of clinical trials over time [35]. A systematic review of 75 studies found that industry-sponsored clinical trials were more likely to report outcomes and conclusions favorable to efficacy compared to studies without industry sponsorship [36]. An observational study analyzing 822 clinical trials related to spinal surgery also found that industry-sponsored trials were more likely to report positive results [37]. The potential conflict of interest inherent in industry-sponsored trials reinforces the need for public and editorial policies requiring full and transparent data disclosure, as well as implementation of strategies to mitigate sponsorship bias.

Use of incidence thresholds or restriction of reporting to pre-specified events alone can obscure adverse reactions that occur less frequently but are clinically relevant. Such practices compromise comprehensive assessment of drug safety and may expose patients to potential risks [38]. Absence of unreported data can affect clinical and regulatory decisions, hinder reproducibility and compromise the reliability of scientific findings [39]. Transparent reporting of all adverse events, regardless of frequency or type, is essential for protecting patient safety and maintaining scientific integrity.

Our findings have important implications for universal health systems like the SUS, which rely on comprehensive and transparent evidence to inform decisions regarding adoption of new treatments, updating of guidelines and monitoring of drug safety [14]. Selective reporting can compromise risk-benefit assessments, influence therapeutic protocols and hinder early detection of adverse events within pharmacovigilance systems [40]. Practices promoting greater transparency are crucial for enhancing patient safety and supporting public policies within the context of the SUS.

In conclusion, selective reporting of adverse renal reactions was frequent in clinical trials involving coxibs and was potentially influenced by editorial policies and research funding. Comprehensive reporting of all outcomes analyzed in clinical studies is essential for greater transparency regarding adverse events, particularly for critical assessment of drug safety and to better inform decision-making and health policy. Robust editorial processes and greater accountability among the stakeholders involved can help ensure the completeness of clinical evidence and the integrity of scientific research.

## SUPPLEMENTARY MATERIAL

SUPPLEMENTARY MATERIAL

## Data Availability

All the data supporting the results of this study has been deposited with the Unicamp Research Data Repository, available at https://doi.org/10.25824/redu/4DSZTA.
